# Supplementary material for: IDP-Bert: Predicting Properties of Intrinsically Disordered Proteins Using Large Language Models
Source: J Phys Chem B. 2024 Nov 25;128(49):12030–7. doi: 10.1021/acs.jpcb.4c02507 (PMC11647883; doi:10.1021/acs.jpcb.4c02507)
Supplement: Supplementary file 1 — jp4c02507_si_001.pdf [file jp4c02507_si_001.pdf]

Supporting Information: IDP-Bert: Predicting Properties of Intrinsically Disordered Proteins (IDP) Using Large Language Models

Parisa Mollaei,[†] Danush Sadasivam,[‡] Chakradhar Guntuboina,[¶] and Amir Barati Farimani[*,†,§,‖]

†*Department of Mechanical Engineering, Carnegie Mellon University, 15213, USA*

‡*Department of Chemical Engineering, Carnegie Mellon University, 15213, USA*

¶*Department of Electrical and Computer Engineering, Carnegie Mellon University, 15213, USA*

§*Department of Biomedical Engineering, Carnegie Mellon University, 15213, USA*

‖*Machine Learning Department, Carnegie Mellon University, 15213, USA*

E-mail: barati@cmu.edu

Experiments

Table S1 presents six experiments showcasing diverse hyperparameters, including the number of epochs, batch size, size and number of hidden layers, number of attention heads, number of layers in the head, and dropout percentage. Following these variations, R^2 results for the train, validation, and test sets are provided. The average R^2 for the test sets serves as the performance metric for the IDP-Bert model in predicting each of the Radius of Gyration,

Table S1: Different experiments conducted with various hyperparameters, each followed by R^2 values for the train, validation, and test sets.

Property	Epochs	Batch size	Hidden layer size	Number of hidden layers	Number of attention heads	Layers in head	Dropout	Train R2	Validation R2	Test R2
Radius of Gyration	5	4	256	8	8	2	0.15	0.9805	0.9864	0.9829
Heat Capacity	5	4	256	8	8	2	0.15	0.9723	0.9607	0.9569
Decorrelation Time	5	4	256	8	8	2	0.15	0.9748	0.9643	0.9560
Radius of Gyration	5	4	512	8	8	2	0.15	0.9877	0.9784	0.9795
Heat Capacity	5	4	512	8	8	2	0.15	0.9799	0.9670	0.9631
Decorrelation Time	5	4	512	8	8	2	0.15	0.9860	0.9662	0.9607
Radius of Gyration	5	4	256	16	16	1	0.15	0.9806	0.9833	0.9823
Heat Capacity	5	4	256	16	16	1	0.15	0.9719	0.9724	0.9665
Decorrelation Time	5	4	256	16	16	1	0.15	0.9728	0.9774	0.9684
Radius of Gyration	5	4	256	16	16	2	0.15	0.9967	0.9879	**0.9899**
Heat Capacity	5	4	256	16	16	2	0.15	0.9794	0.9733	**0.9689**
Decorrelation Time	5	4	256	16	16	2	0.15	0.9866	0.9799	**0.9687**

end-to-end Decorrelation Time, and Heat Capacity properties.

Contribution of sequence length in predicting properties

To assess the contribution of sequence length in predicting properties, as well as to examine the importance of sequence information, we conducted a modified training procedure. We modified the dataset so that for each protein sequence, we retained the length but replaced the actual sequence with a string of 'A's, effectively removing any meaningful sequence information. The performance of the model trained on this modified dataset is reported in

Table S2.

We observed that the R^2 scores were significantly lower, indicating that while length does have some correlation with the properties, it is insufficient to achieve high prediction R^2 scores. This highlights the importance of the specific sequence information in making accurate predictions.

Table S2: Training, Validation, and Testing R^2 values for Radius of Gyration, Heat Capacity, and Decorrelation Time when the amino acid sequences were replaced with a string of 'A's.

**Property**	**Train R2**	**Validation R2**	**Test R2**
Radius of Gyration	0.5184	0.7337	0.7436
Heat Capacity	0.5365	0.5636	0.6448
Decorrelation Time	0.2519	0.2188	0.3384

Protein taxonomy of the dataset

Similarity search of the sequences in the dataset was performed on the UniProt database, and the taxonomy information of each of the similarity hits were noted in a csv file. This taxonomy information was obtained using the NCBI Entrez API available within the BioPython library. For each of the similarity hits, the UniProt ID, the similarity fraction, and the taxonomy information is provided in the csv file named "taxonomy_results.csv".
